# Supplementary material for: Metal-Free Catalyzed Oxidation/Decarboxylative [3+2] Cycloaddition Sequences of 3-Formylchromones to Access Pyrroles with Anti-Cancer Activity
Source: Molecules. 2023 Nov 15;28(22):7602. doi: 10.3390/molecules28227602 (PMC10673291; doi:10.3390/molecules28227602)

Metal-free catalyzed oxidation/decarboxylative [3+2] cycloaddition sequences of 3-formylchromones to access pyrroles with anti-cancer activity

Xue Li [1,2], Xing-Yu Chen [1], Bing-Ying Fan [1], Qun Yu [1], Jie Lei [1,3,4,*], Zhi-Gang Xu [1,*] and Zhong-Zhu Chen [1,*]

[1] *College of Pharmacy, National & Local Joint Engineering Research Center of Targeted and Innovative Therapeutics, IATTI, Chongqing University of Arts and Sciences, Chongqing 402160, China; lxshirley@cqwu.edu.cn (X.L.); 15320378927@163.com (X.-Y.C.); 15310400564@163.com (B.-Y.F.); 15084401382@163.com (Q.Y.)*

[2] *Chongqing Key laboratory of Natural Product Synthesis and Drug Research, School of Pharmaceutical Sciences, Chongqing University, Chongqing, 400044, China*

[3] *Institute of Bioorganic & Medicinal Chemistry, School of Chemistry and Chemical Engineering, Southwest University, Chongqing, 400715, China*

[4] *Chongqing Academy of Chinese Materia Medica, Chongqing, 400067, China*

* *Correspondence: jlei@cqwu.edu.cn (J.L.); xzg@cqwu.edu.cn (Z.-G.X.); 18883138277@163.com (Z.-Z.C.)*

Table of Contents **Page**

Crystal data of **3g**……………………………………………………………S2-S6

NMR Figures of Products...S7-S23

Table S1 Crystal data and structure refinement for 3g.	
Identification code	**3g**
Empirical formula	$C_{18}H_{14}ClNO_4S$
Formula weight	375.81
Temperature/K	273.00
Crystal system	monoclinic
Space group	$P2_1/n$
a/Å	5.2121(5)
b/Å	30.159(4)
c/Å	10.9824(13)
α/°	90
β/°	92.693(4)
γ/°	90
Volume/Å^3	1724.4(3)
Z	4
ρ_{calc}g/cm^3	1.448
μ/mm^{-1}	0.365
F(000)	776.0
Crystal size/mm^3	0.21 × 0.16 × 0.1
Radiation	MoKα (λ = 0.71073)
2Θ range for data collection/°	4.592 to 56.62
Index ranges	$-6 \leq h \leq 5$, $-40 \leq k \leq 40$, $-14 \leq l \leq 14$
Reflections collected	26265
Independent reflections	4250 [R_{int} = 0.0448, R_{sigma} = 0.0294]
Data/restraints/parameters	4250/0/228
Goodness-of-fit on F^2	1.034
Final R indexes [I>=2σ (I)]	R_1 = 0.0433, wR_2 = 0.1012
Final R indexes [all data]	R_1 = 0.0618, wR_2 = 0.1128
Largest diff. peak/hole / e Å^{-3}	0.27/-0.32

Table S2 Fractional Atomic Coordinates (×10^4) and Equivalent Isotropic Displacement Parameters (Å^2×10^3) for 3g. U_{eq} is defined as 1/3 of the trace of the orthogonalised U_{IJ} tensor.

**Atom**	*x*	*y*	*z*	**U(eq)**
S1	8267.4(9)	6954.7(2)	2661.7(4)	38.43(13)
Cl1	2745.9(14)	9500.1(2)	4917.0(6)	75.8(2)
O3	468(3)	8102.5(4)	757.1(11)	44.8(3)
O1	10083(3)	6979.1(5)	3684.5(13)	48.1(3)
O2	9144(3)	6975.7(5)	1439.8(13)	54.3(4)
O4	-3190(3)	8680.9(5)	856.9(14)	54.1(4)
N1	5263(3)	7535.5(5)	3876.2(13)	39.2(4)
C13	440(4)	8635.2(6)	2316.8(16)	35.8(4)
C10	3204(4)	7928.9(6)	2430.1(15)	34.8(4)
C8	6070(4)	7385.4(6)	2773.0(16)	36.5(4)
C12	1345(3)	8214.7(6)	1782.1(15)	34.7(4)
C9	4816(4)	7619.9(6)	1859.6(16)	37.5(4)
C14	1800(4)	8842.7(6)	3285.7(17)	41.7(4)
C11	3543(4)	7861.6(6)	3677.5(16)	38.9(4)
C5	6464(4)	6463.6(6)	2803.3(17)	40.0(4)
C18	-1772(4)	8846.3(6)	1804.3(18)	42.4(4)
C15	961(4)	9240.0(7)	3744(2)	49.3(5)
C4	7025(4)	6176.4(7)	3759(2)	52.5(5)
C17	-2587(4)	9244.6(7)	2297(2)	55.1(5)
C16	-1229(4)	9440.8(7)	3257(2)	57.9(6)
C6	4485(5)	6371.4(8)	1965(2)	62.0(6)
C2	3606(5)	5693.4(8)	3024(3)	69.0(7)
C3	5586(5)	5792.7(8)	3856(3)	65.7(7)
C7	3080(5)	5987.6(9)	2091(3)	80.0(9)
C1	1991(7)	5278.0(10)	3170(4)	113.9(14)

Table S3 Anisotropic Displacement Parameters (Å2×103) for 3g. The Anisotropic displacement factor exponent takes the form: -2π2[h2a*2U11+2hka*b*U12+…].

Atom	U_{11}	U_{22}	U_{33}	U_{23}	U_{13}	U_{12}
S1	37.2(2)	42.0(3)	35.6(2)	4.43(18)	-3.45(18)	3.38(18)
Cl1	84.1(5)	62.6(4)	78.8(4)	-38.9(3)	-16.7(3)	3.8(3)
O3	59.9(9)	40.8(7)	32.6(7)	-6.9(5)	-9.4(6)	6.8(6)
O1	42.4(8)	51.4(8)	49.1(8)	7.2(6)	-13.3(6)	-3.3(6)
O2	55.0(9)	67.7(10)	40.7(8)	8.2(7)	8.6(7)	13.3(7)
O4	53.6(9)	51.5(9)	55.5(9)	-7.0(7)	-17.4(7)	8.5(7)
N1	50.2(9)	38.8(8)	28.1(7)	4.9(6)	-4.0(6)	-1.4(7)
C13	41.1(10)	31.4(9)	34.8(9)	-1.9(7)	0.7(7)	0.2(7)
C10	44.9(10)	30.0(8)	29.1(8)	0.0(6)	-1.9(7)	-1.0(7)
C8	42.0(10)	35.3(9)	32.0(9)	2.3(7)	-1.8(7)	0.0(7)
C12	42.9(10)	30.7(8)	30.3(9)	-0.6(7)	0.8(7)	-1.8(7)
C9	46.8(10)	36.7(9)	28.7(8)	1.0(7)	-1.2(7)	2.5(8)
C14	46.6(11)	36.6(9)	41.4(10)	-5.9(8)	-2.8(8)	1.3(8)
C11	50.8(11)	34.6(9)	31.2(9)	0.2(7)	-0.1(8)	2.0(8)
C5	38.0(10)	38.3(9)	43.1(10)	-5.7(8)	-2.7(8)	5.6(7)
C18	42.4(10)	37.5(10)	47.1(11)	-0.8(8)	-1.4(8)	0.2(8)
C15	55.1(12)	40.8(10)	51.9(12)	-14.8(9)	1.1(10)	-2.3(9)
C4	58.4(13)	44.8(11)	53.2(12)	2.1(9)	-8.1(10)	-2.5(9)
C17	46.5(12)	45.4(11)	72.9(15)	-7.2(10)	-3.7(11)	11.7(9)
C16	55.8(13)	41.5(11)	76.6(16)	-18.9(10)	4.9(11)	7.7(9)
C6	56.1(14)	59.5(14)	68.0(15)	-5.9(11)	-22.5(11)	0.9(11)
C2	51.9(14)	42.2(12)	114(2)	-20.6(13)	13.8(14)	-4.0(10)
C3	73.5(17)	43.0(12)	81.1(18)	6.2(11)	7.6(13)	-0.7(11)
C7	56.5(15)	68.0(17)	113(2)	-25.8(16)	-22.5(15)	-7.2(13)
C1	82(2)	54.5(17)	207(4)	-24(2)	26(2)	-20.0(15)

Table S4 Bond Lengths for 3g.

Atom	Atom	Length/Å	Atom	Atom	Length/Å
S1	O1	1.4363(14)	C10	C11	1.388(2)
S1	O2	1.4391(15)	C8	C9	1.368(2)
S1	C8	1.7398(19)	C14	C15	1.379(3)
S1	C5	1.765(2)	C5	C4	1.382(3)
Cl1	C15	1.740(2)	C5	C6	1.379(3)
O3	C12	1.242(2)	C18	C17	1.392(3)
O4	C18	1.344(2)	C15	C16	1.378(3)
N1	C8	1.378(2)	C4	C3	1.386(3)
N1	C11	1.342(2)	C17	C16	1.376(3)
C13	C12	1.484(2)	C6	C7	1.380(4)
C13	C14	1.398(2)	C2	C3	1.378(4)
C13	C18	1.411(3)	C2	C7	1.373(4)
C10	C12	1.457(2)	C2	C1	1.522(4)
C10	C9	1.420(2)			

Table S5 Bond Angles for 3g.

Atom	Atom	Atom	Angle/°	Atom	Atom	Atom	Angle/°
O1	S1	O2	120.03(9)	C15	C14	C13	120.56(19)
O1	S1	C8	108.58(9)	N1	C11	C10	108.71(16)
O1	S1	C5	107.95(9)	C4	C5	S1	119.90(15)
O2	S1	C8	105.75(9)	C6	C5	S1	119.70(17)
O2	S1	C5	108.23(9)	C6	C5	C4	120.4(2)
C8	S1	C5	105.39(9)	O4	C18	C13	123.43(17)
C11	N1	C8	109.10(15)	O4	C18	C17	117.02(19)
C14	C13	C12	121.63(17)	C17	C18	C13	119.55(19)
C14	C13	C18	118.50(16)	C14	C15	Cl1	119.61(17)
C18	C13	C12	119.79(16)	C16	C15	Cl1	119.58(16)

C9	C10	C12	124.50(16)
C11	C10	C12	128.28(17)
C11	C10	C9	106.79(16)
N1	C8	S1	122.44(13)
C9	C8	S1	128.89(14)
C9	C8	N1	108.65(16)
O3	C12	C13	118.77(16)
O3	C12	C10	119.31(16)
C10	C12	C13	121.92(15)
C8	C9	C10	106.73(16)

C16	C15	C14	120.77(19)
C5	C4	C3	119.0(2)
C16	C17	C18	120.9(2)
C17	C16	C15	119.69(19)
C5	C6	C7	119.1(2)
C3	C2	C1	120.6(3)
C7	C2	C3	118.1(2)
C7	C2	C1	121.2(3)
C2	C3	C4	121.5(2)
C2	C7	C6	121.9(2)

Figure S1. ^{1}H NMR and ^{13}C NMR spectra of **3a**.

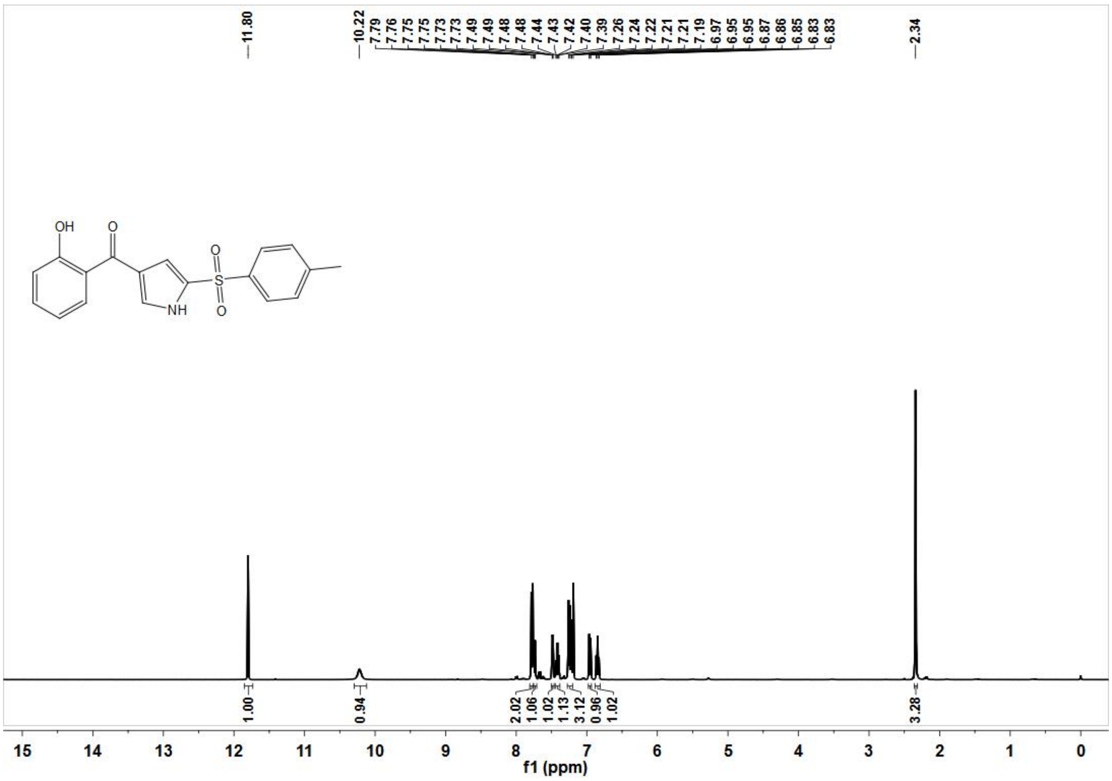

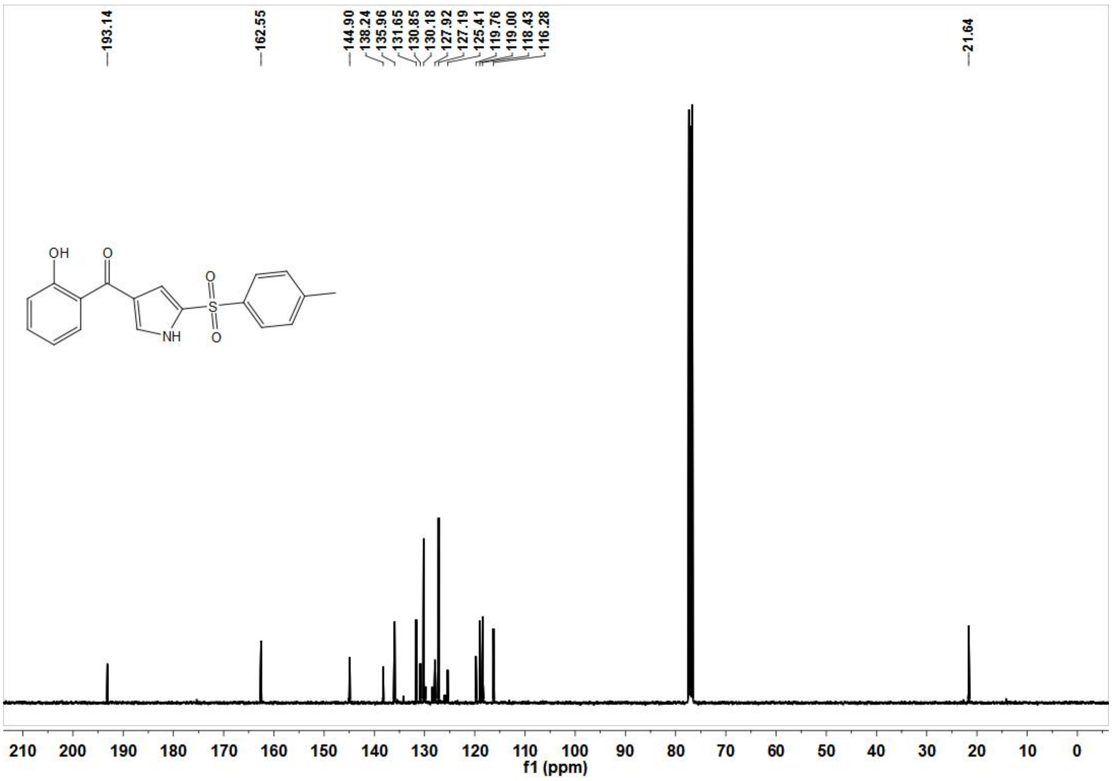

Figure S2. ^{1}H NMR and ^{13}C NMR spectra of **3b.**

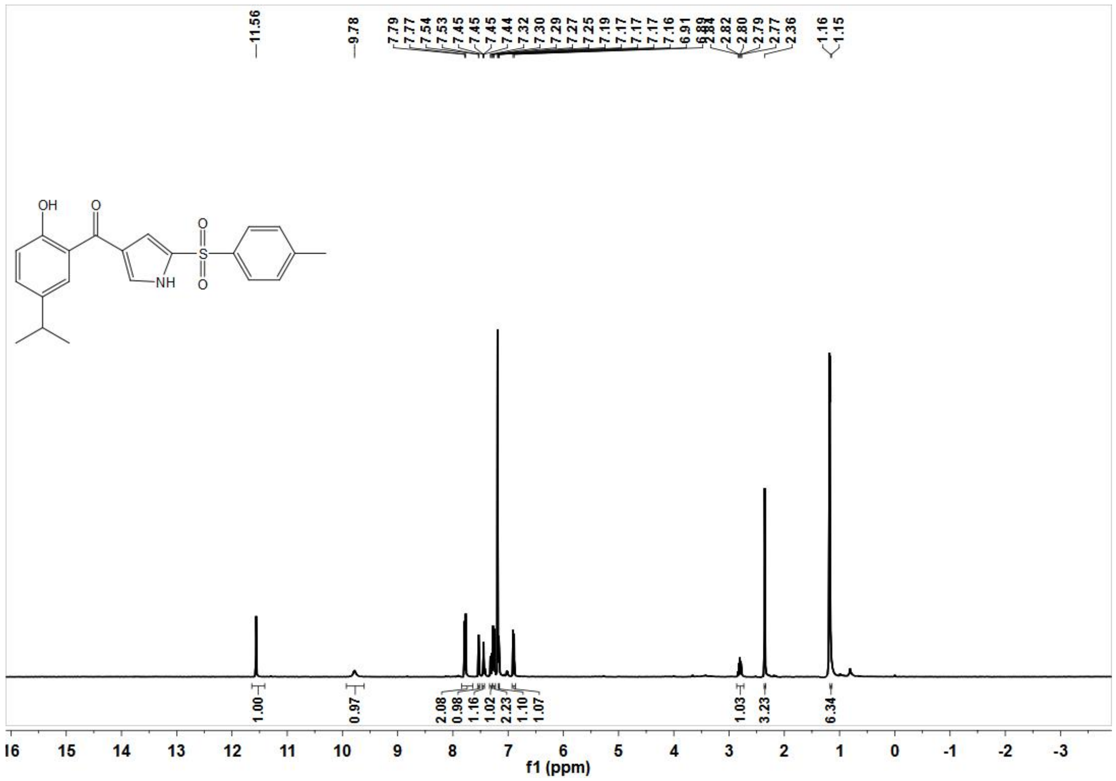

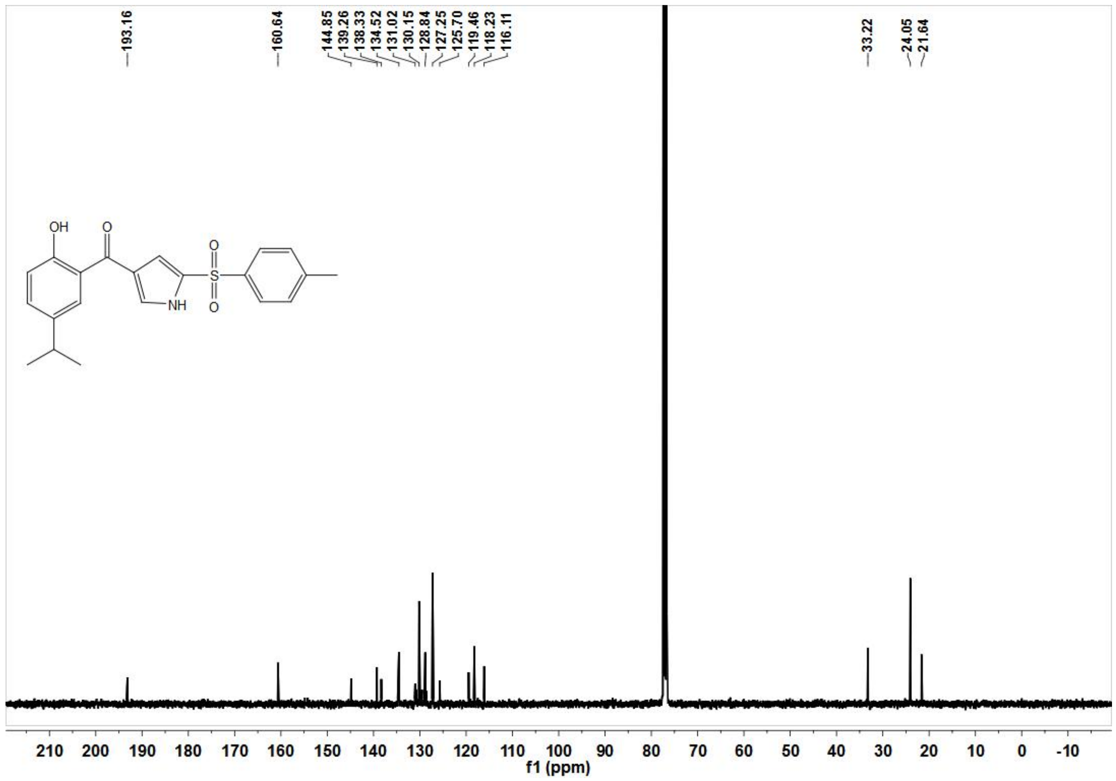

Figure S3. ^{1}H NMR and ^{13}C NMR spectra of **3c.**

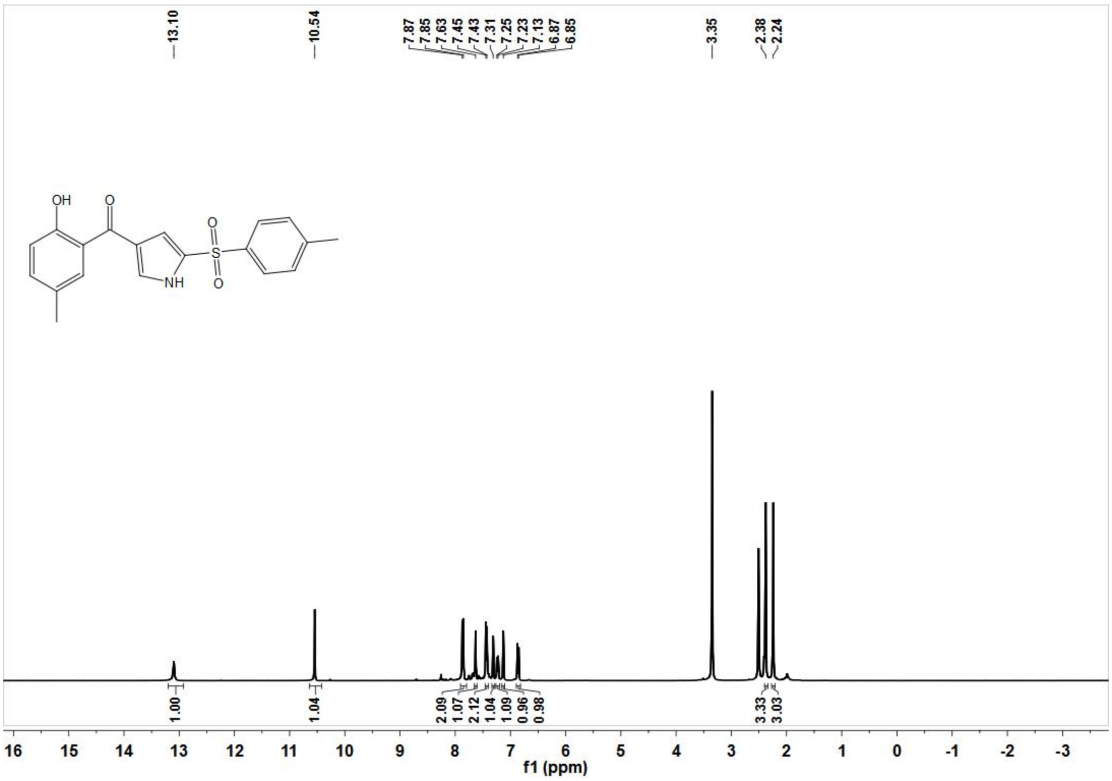

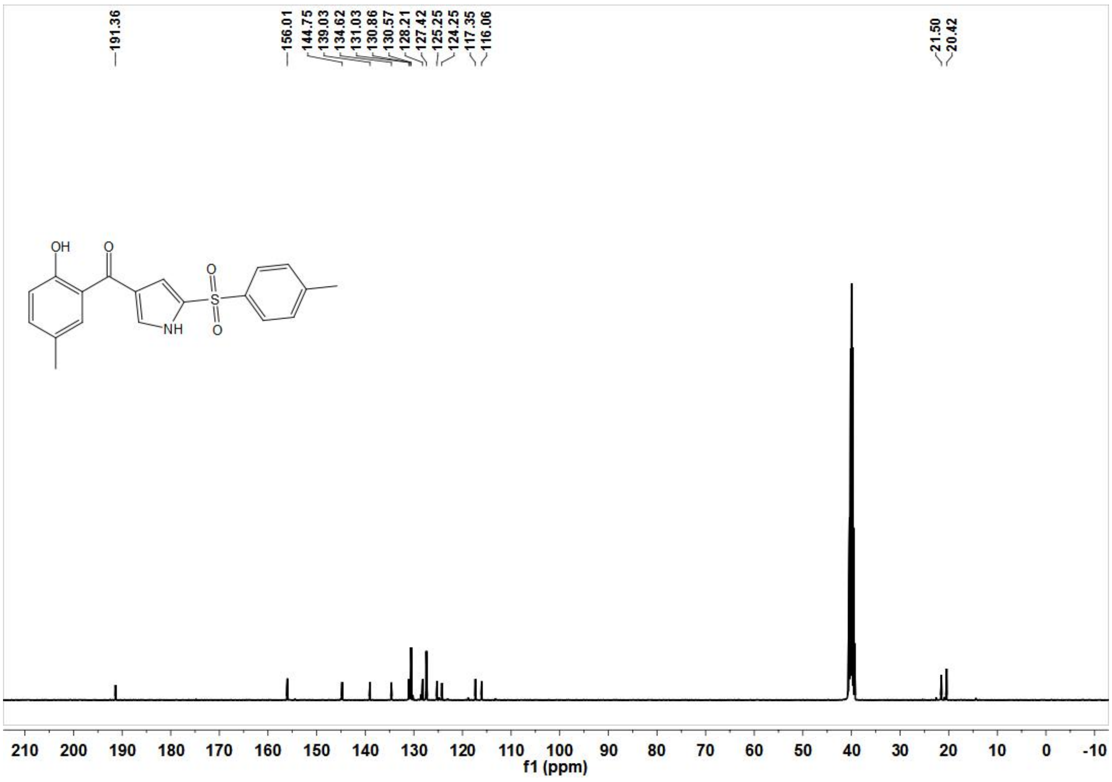

Figure S4. ^{1}H NMR and ^{13}C NMR spectra of **3d.**

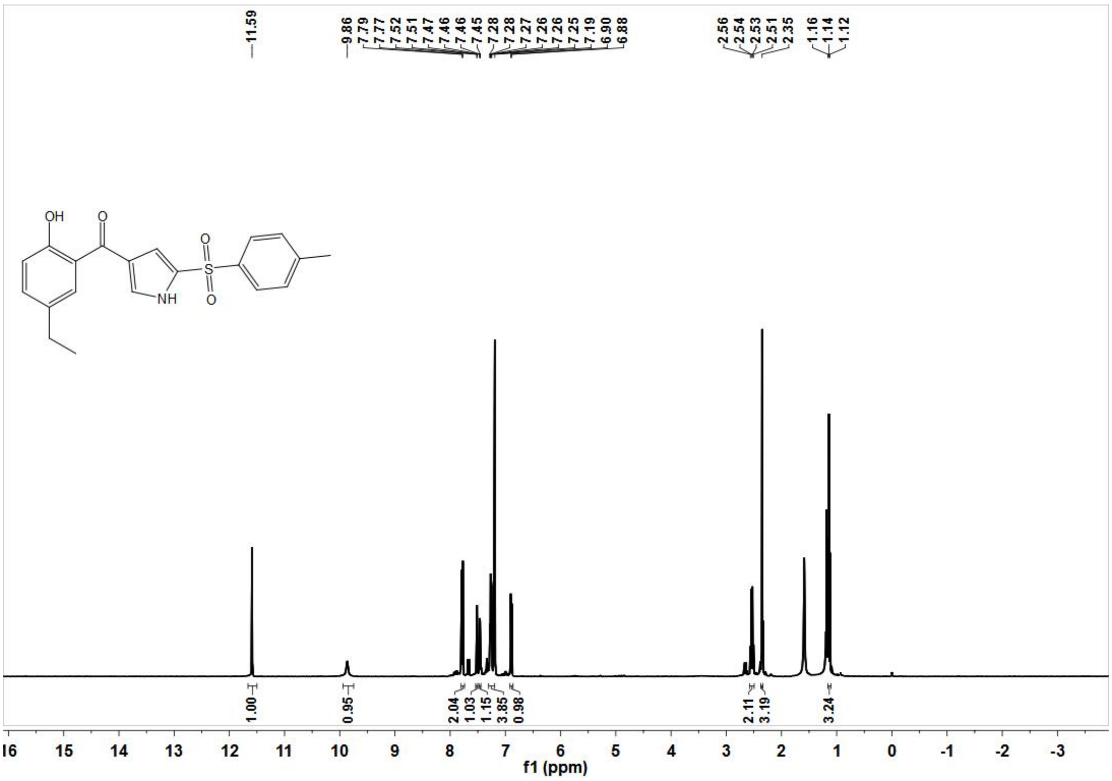

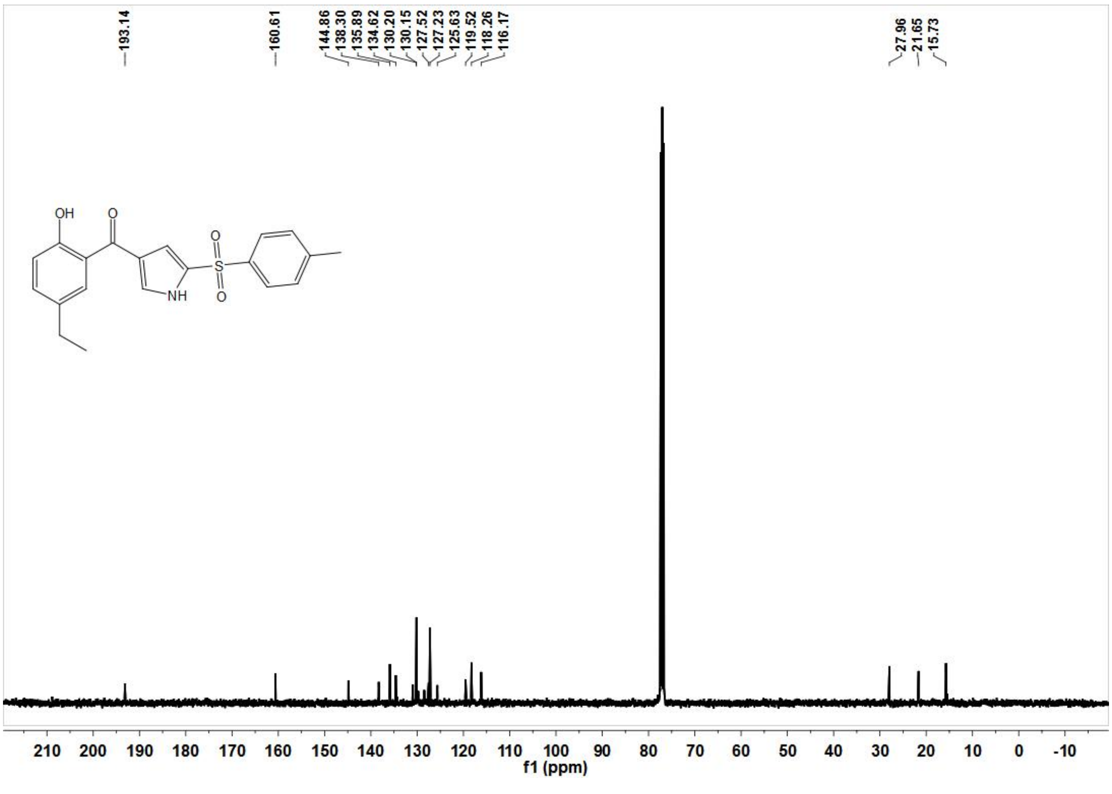

Figure S5. ^{1}H NMR and ^{13}C NMR spectra of **3e.**

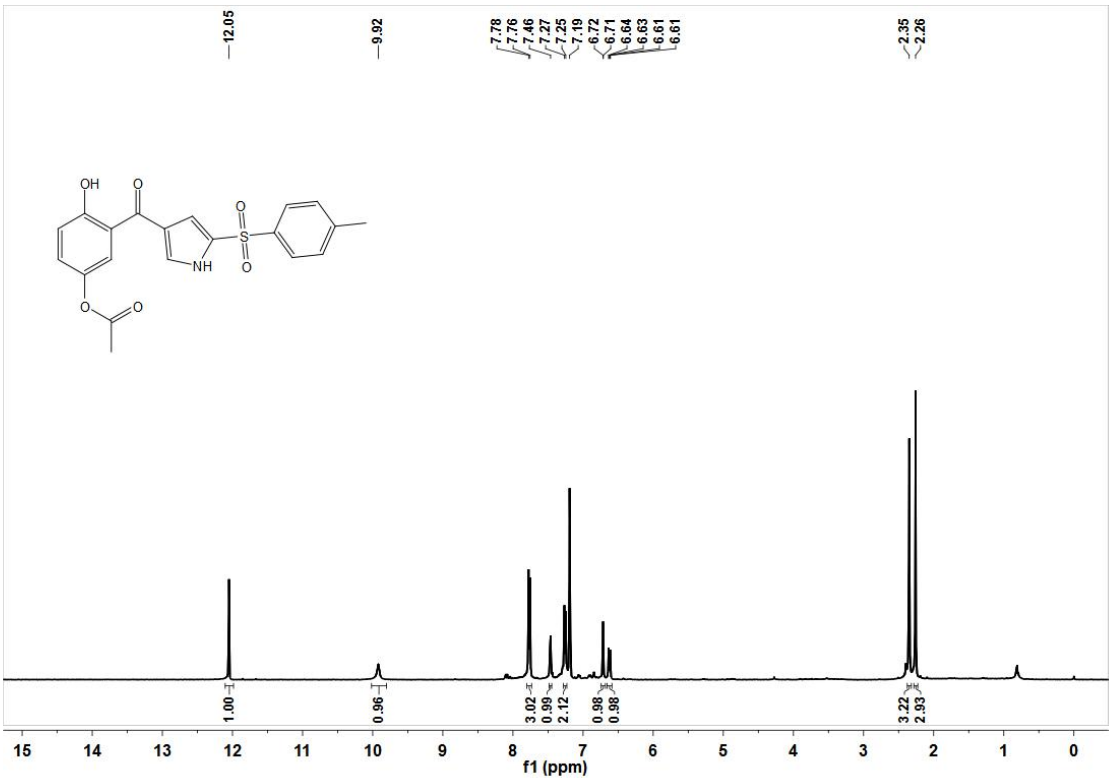

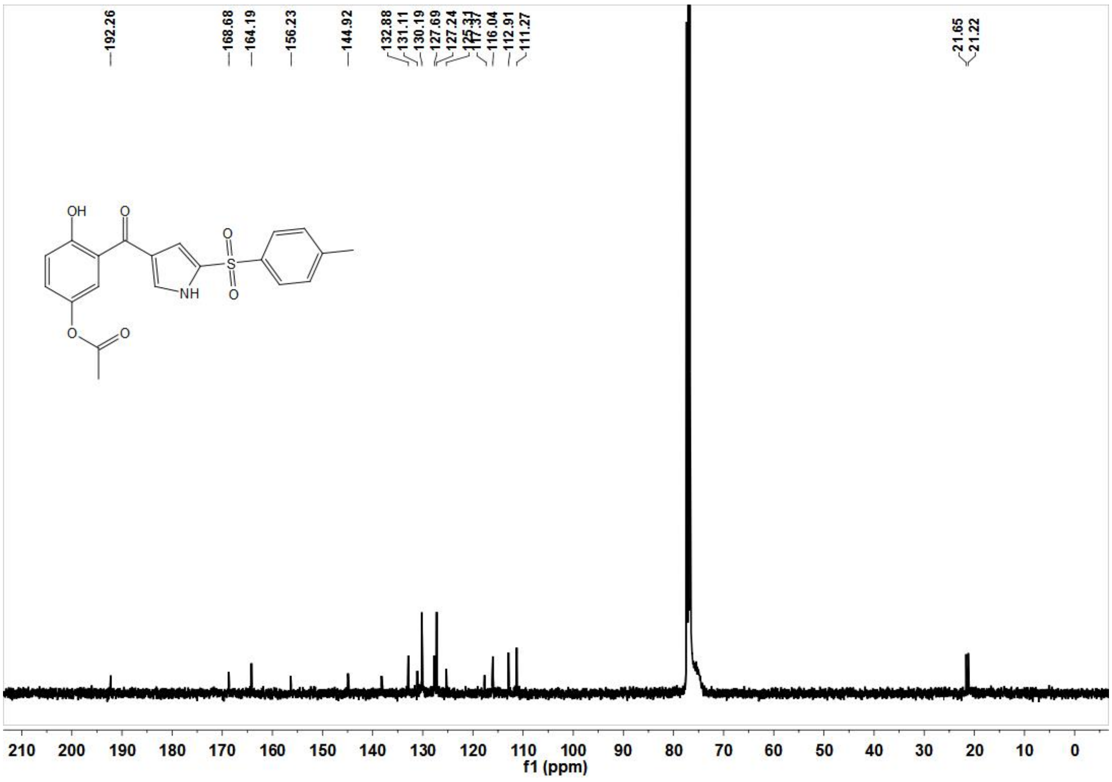

Figure S6. ^{1}H NMR and ^{13}C NMR spectra of **3f.**

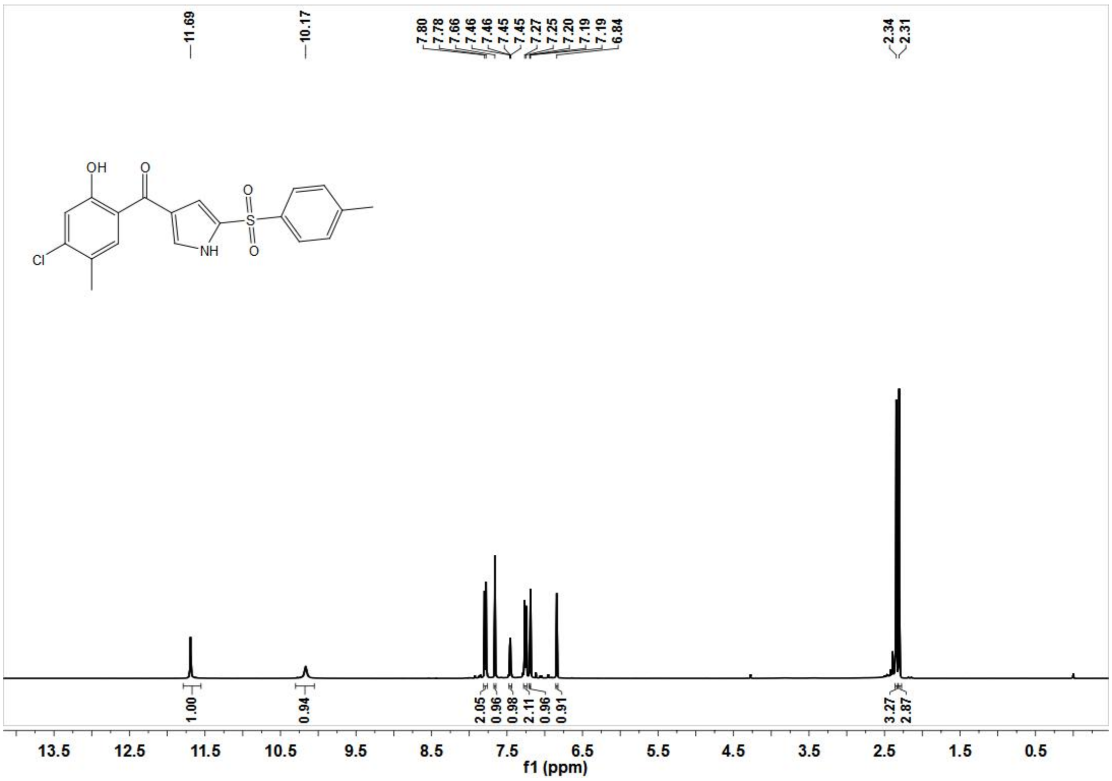

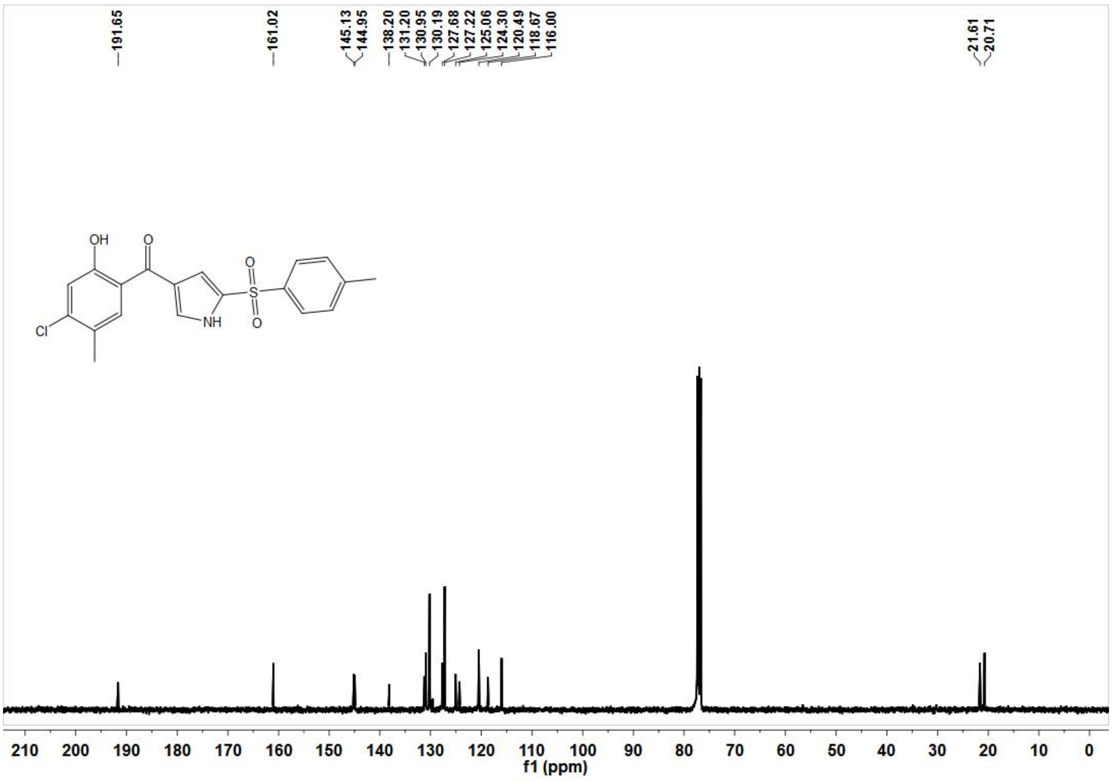

Figure S7. ^{1}H NMR and ^{13}C NMR spectra of **3g.**

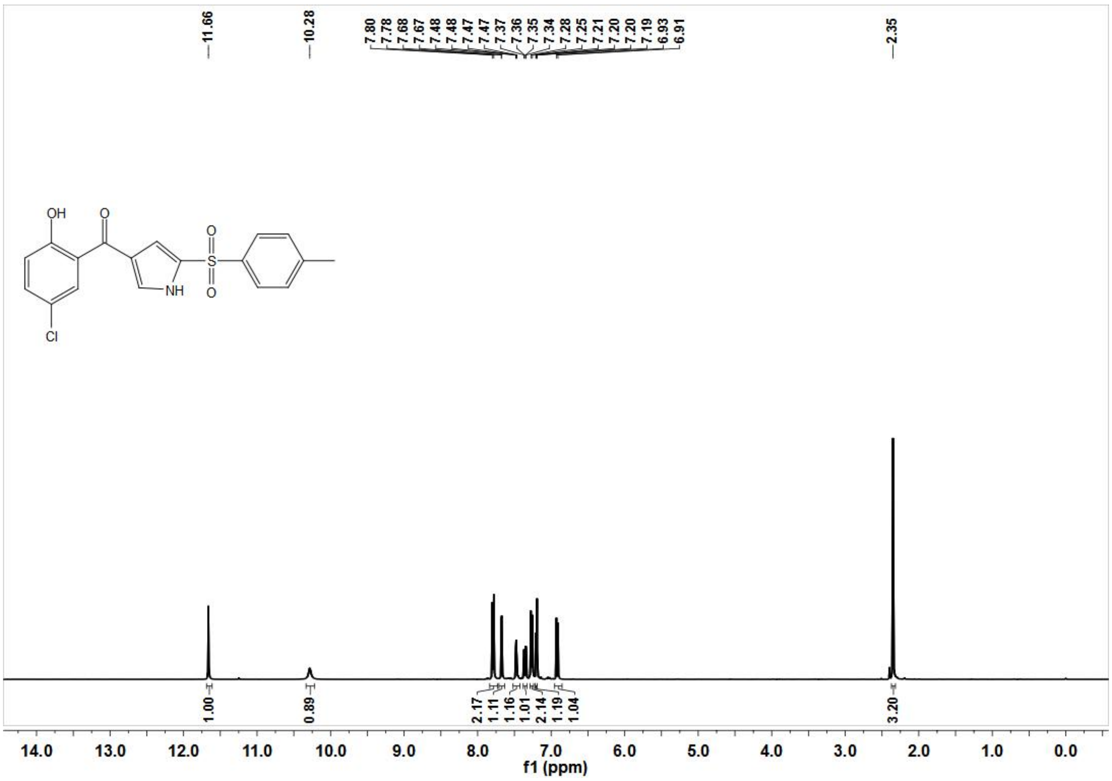

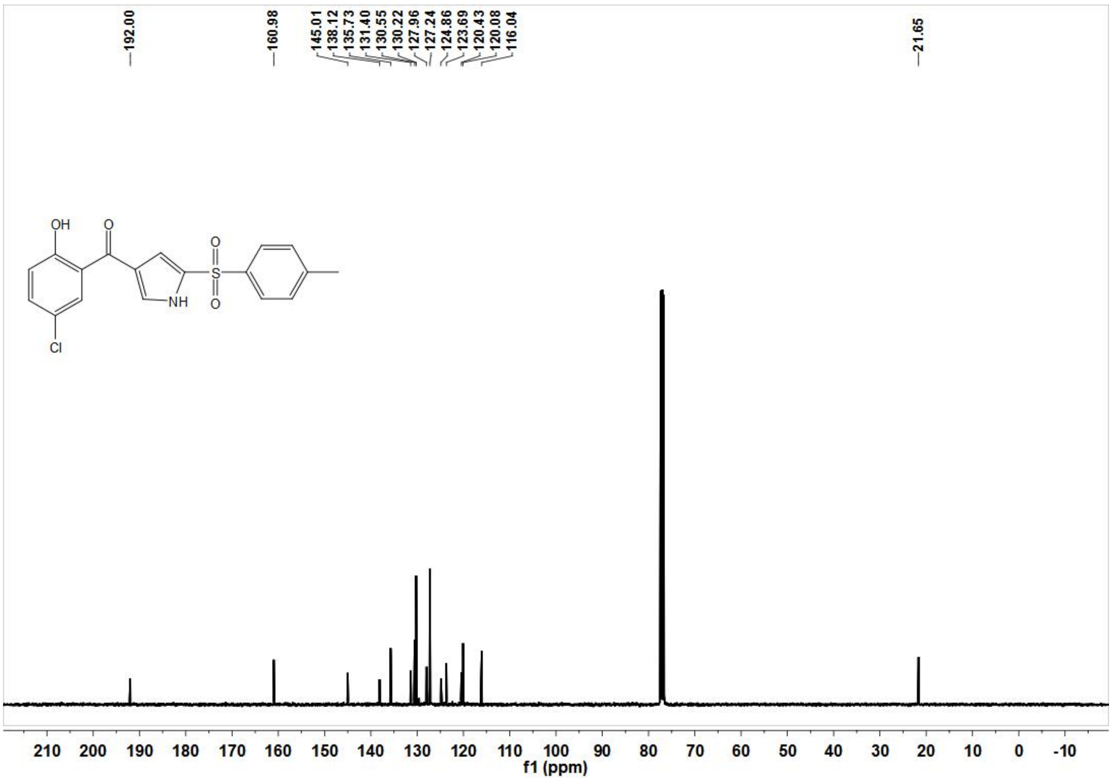

Figure S8. ^{1}H NMR and ^{13}C NMR spectra of **3h.**

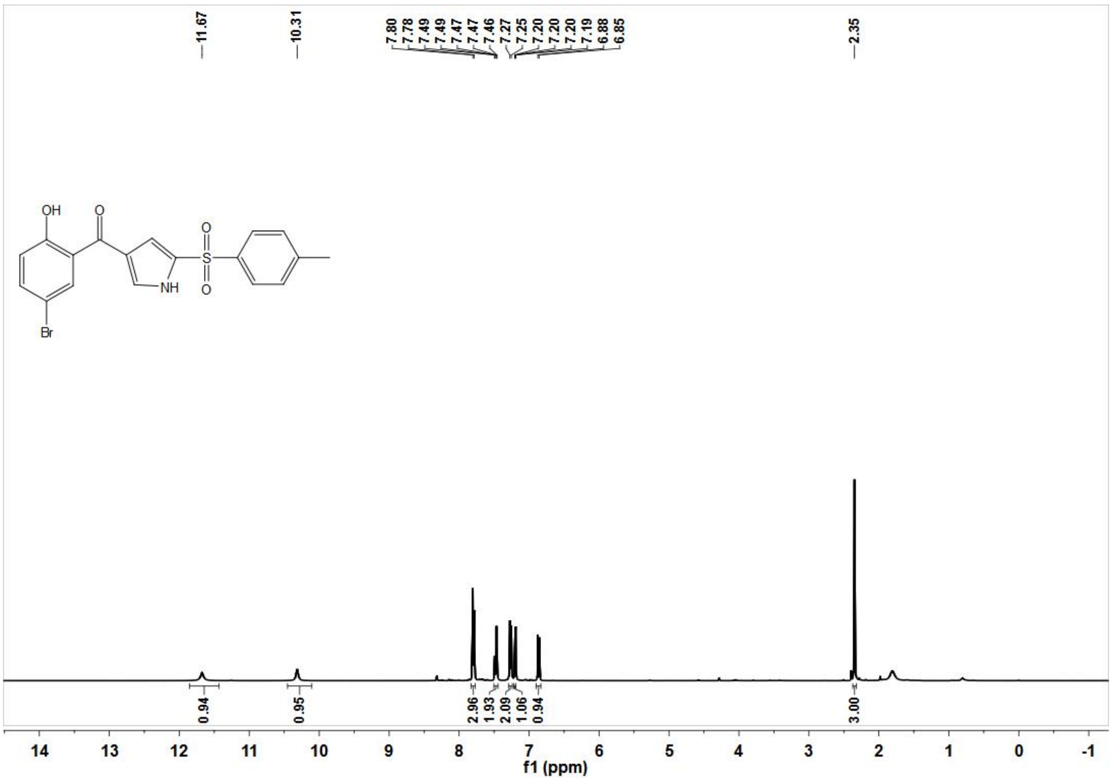

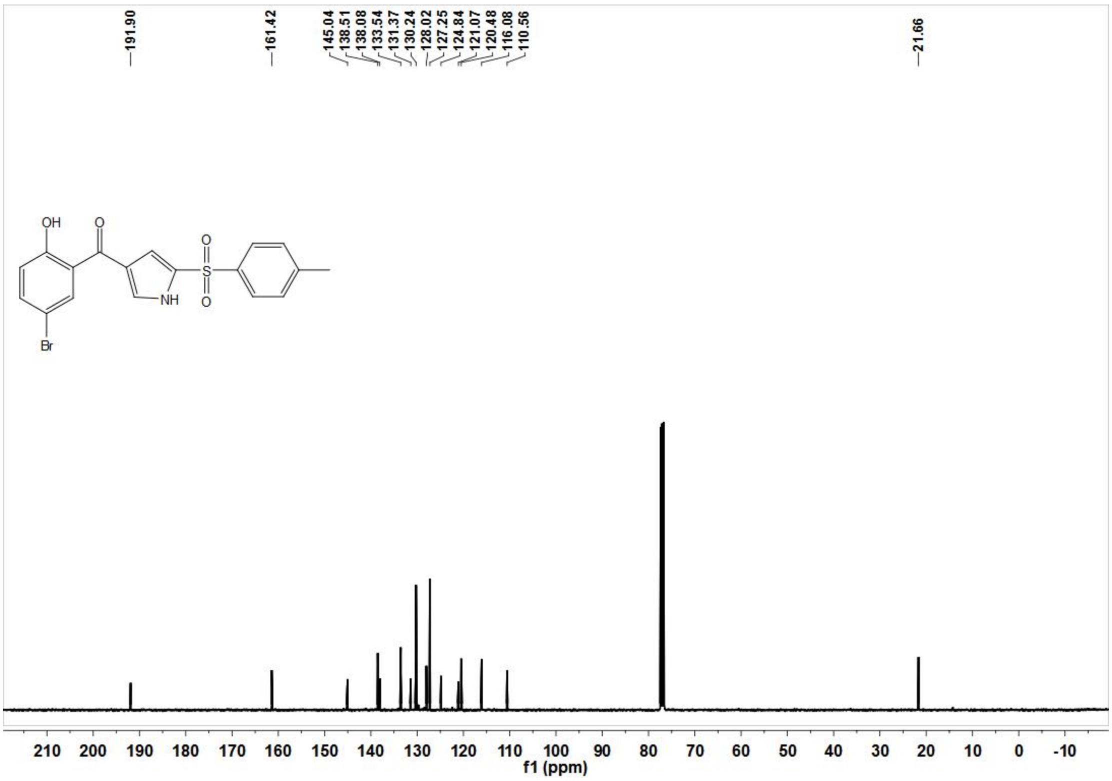

Figure S9. ^{1}H NMR and ^{13}C NMR spectra of **3i.**

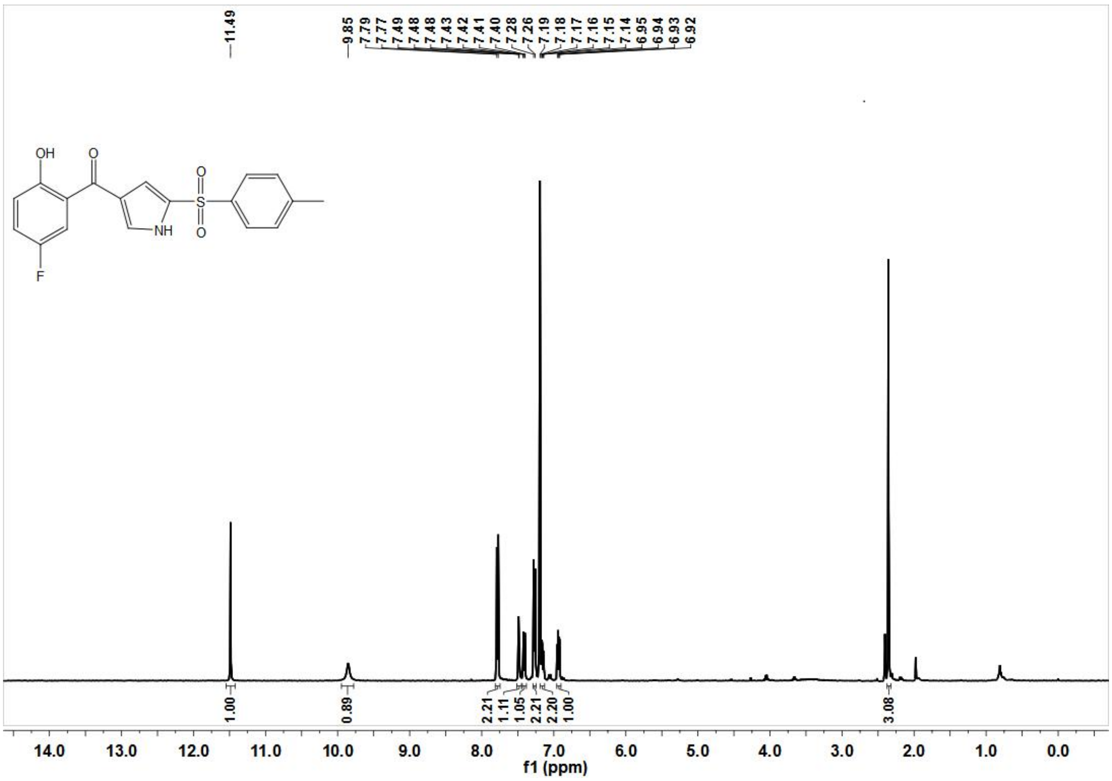

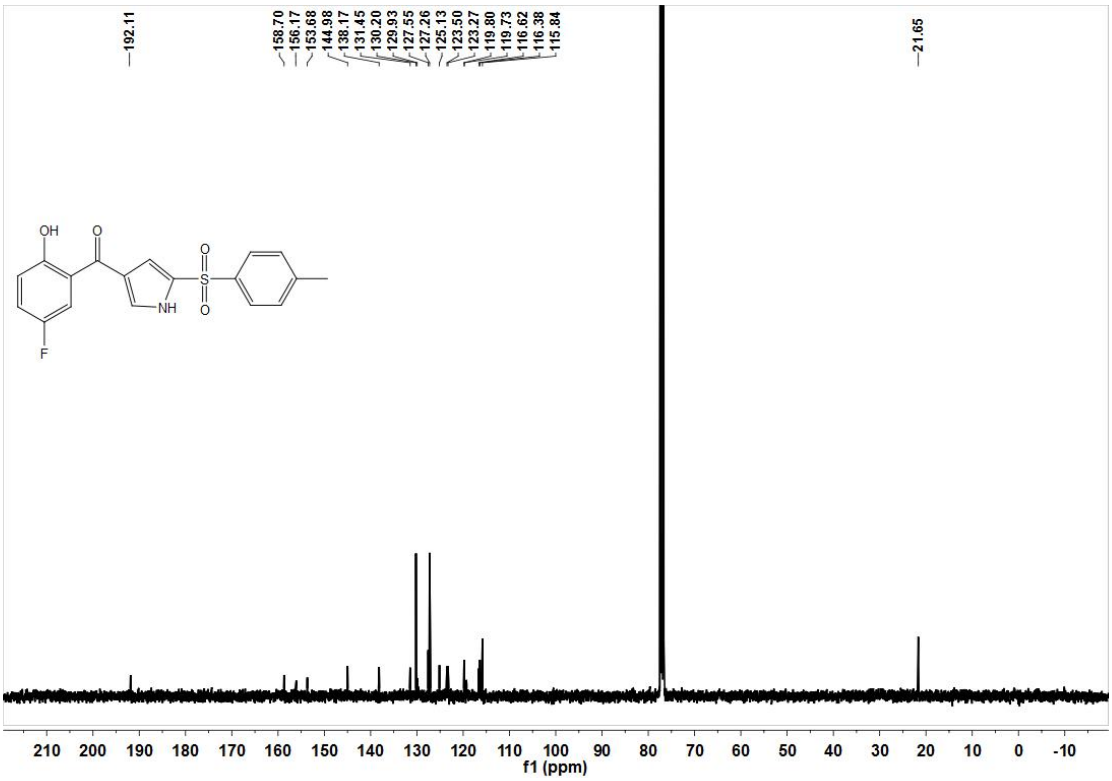

Figure S10. ^{1}H NMR and ^{13}C NMR spectra of **3j.**

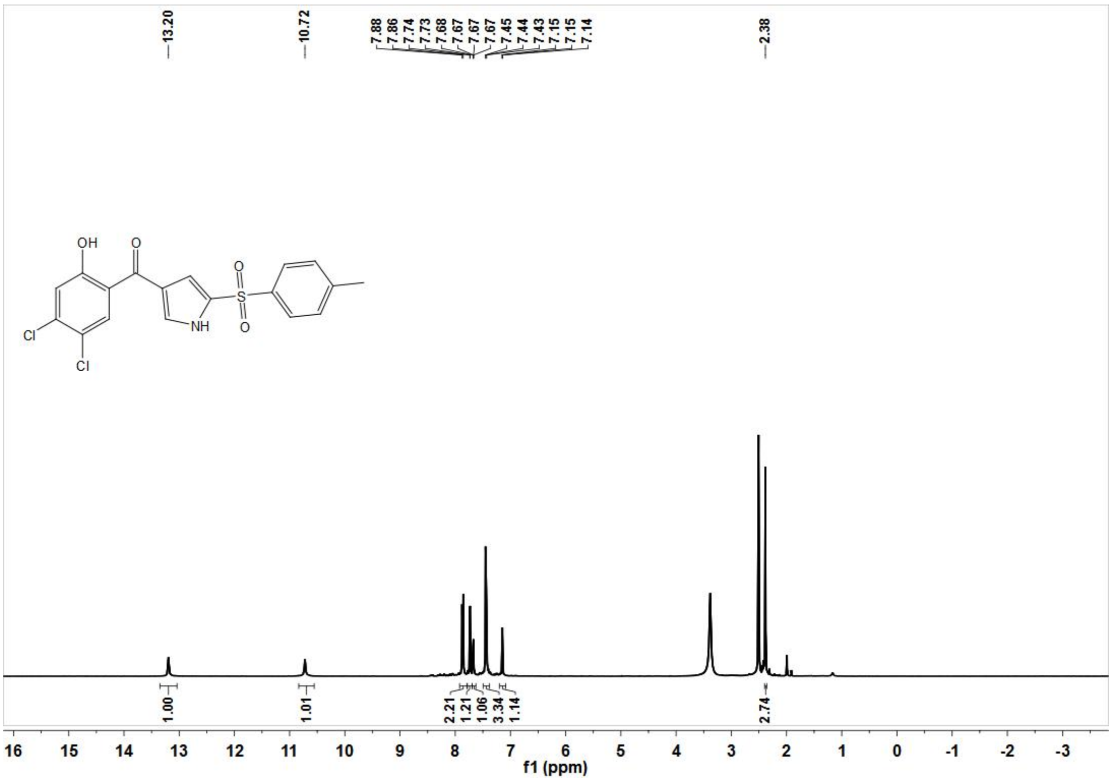

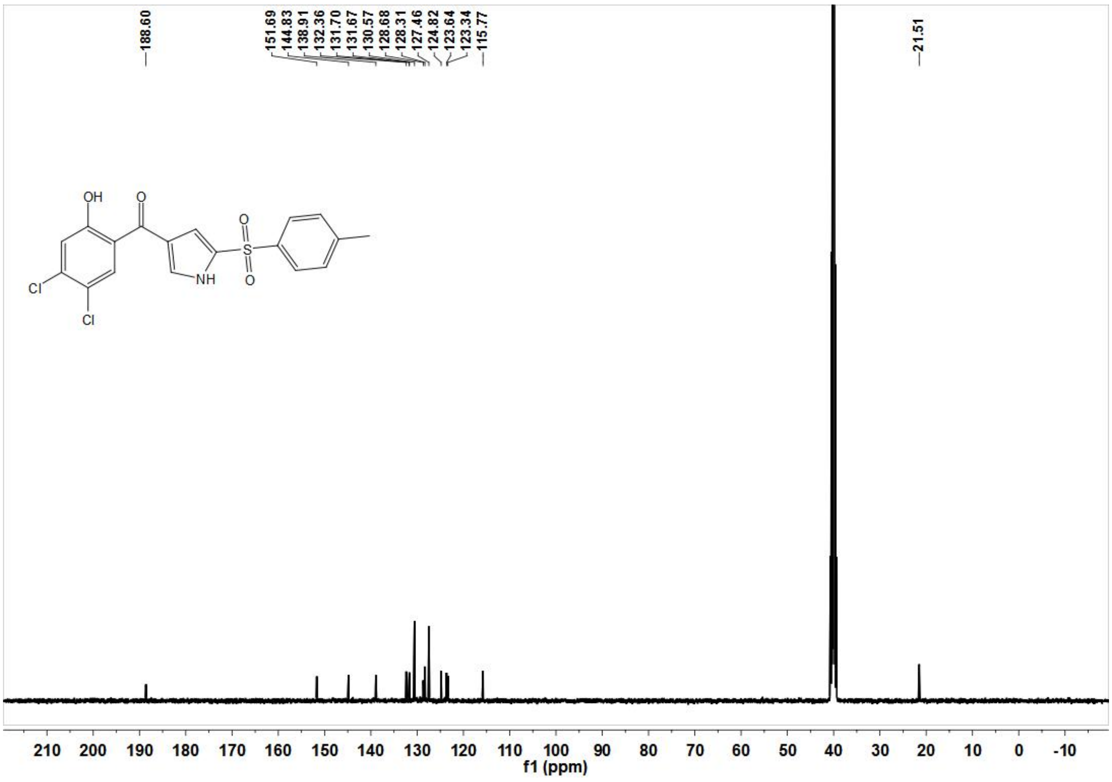

Figure S11. ^{1}H NMR and ^{13}C NMR spectra of **3k.**

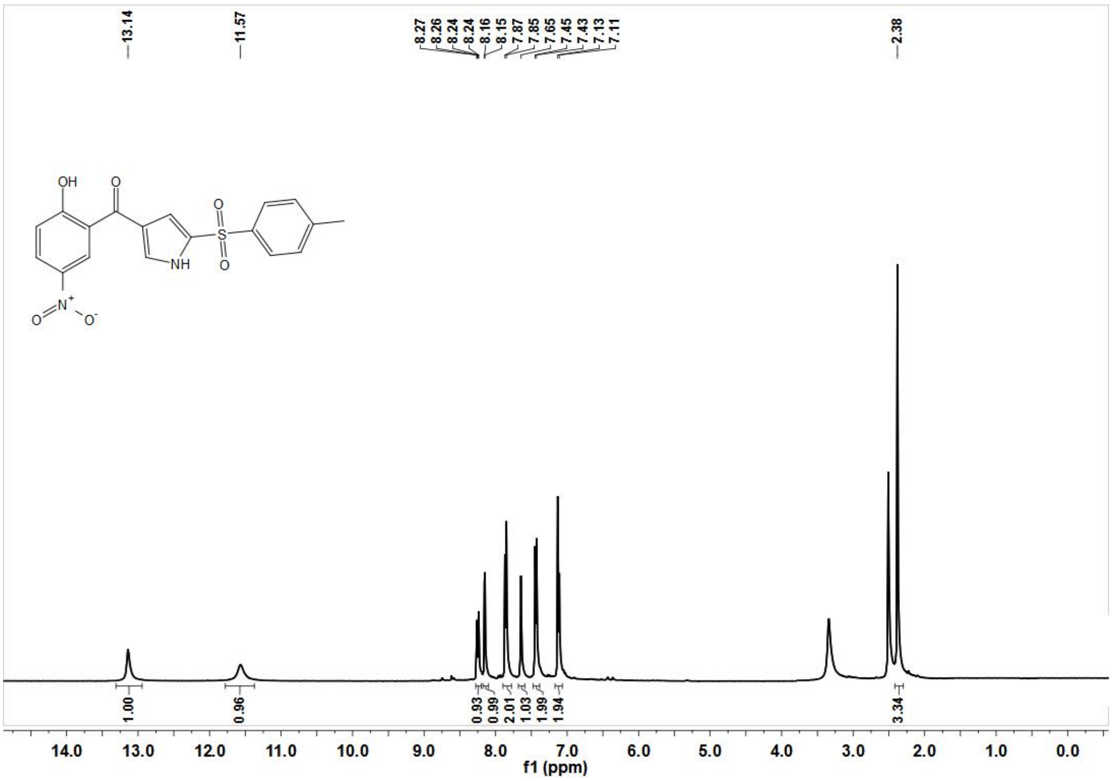

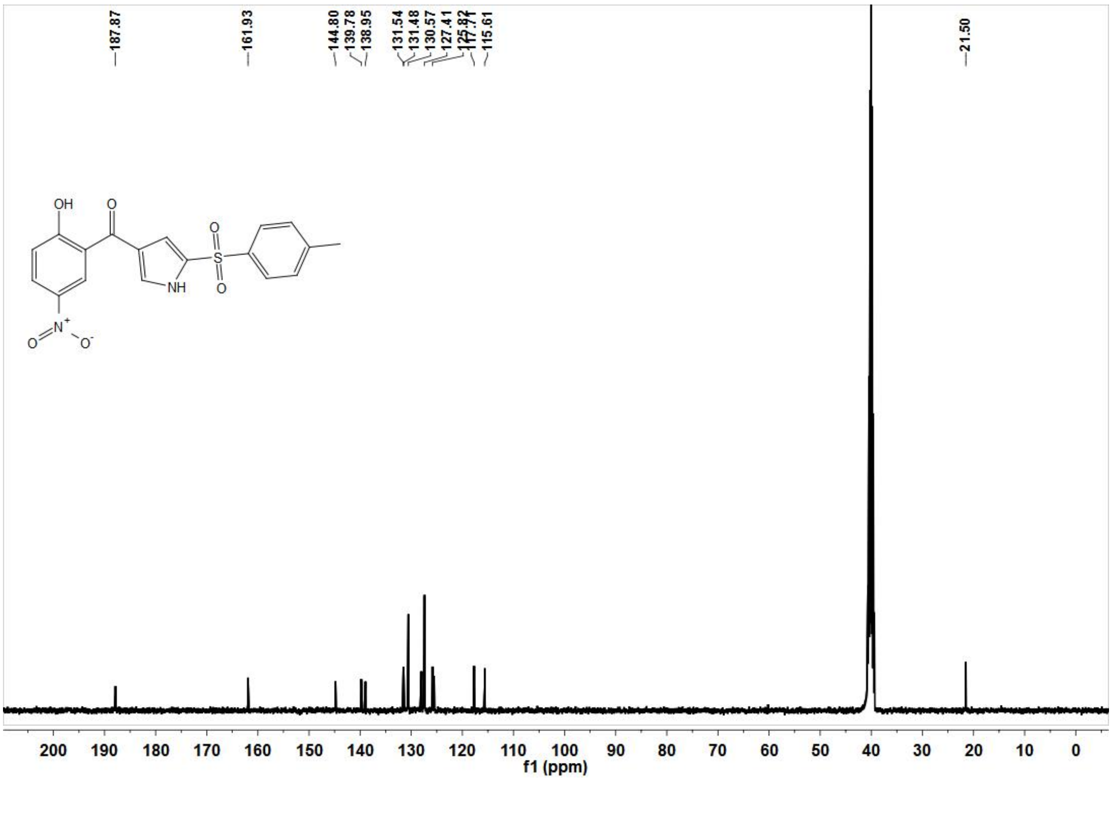

Figure S12. ^{1}H NMR and ^{13}C NMR spectra of **3l.**

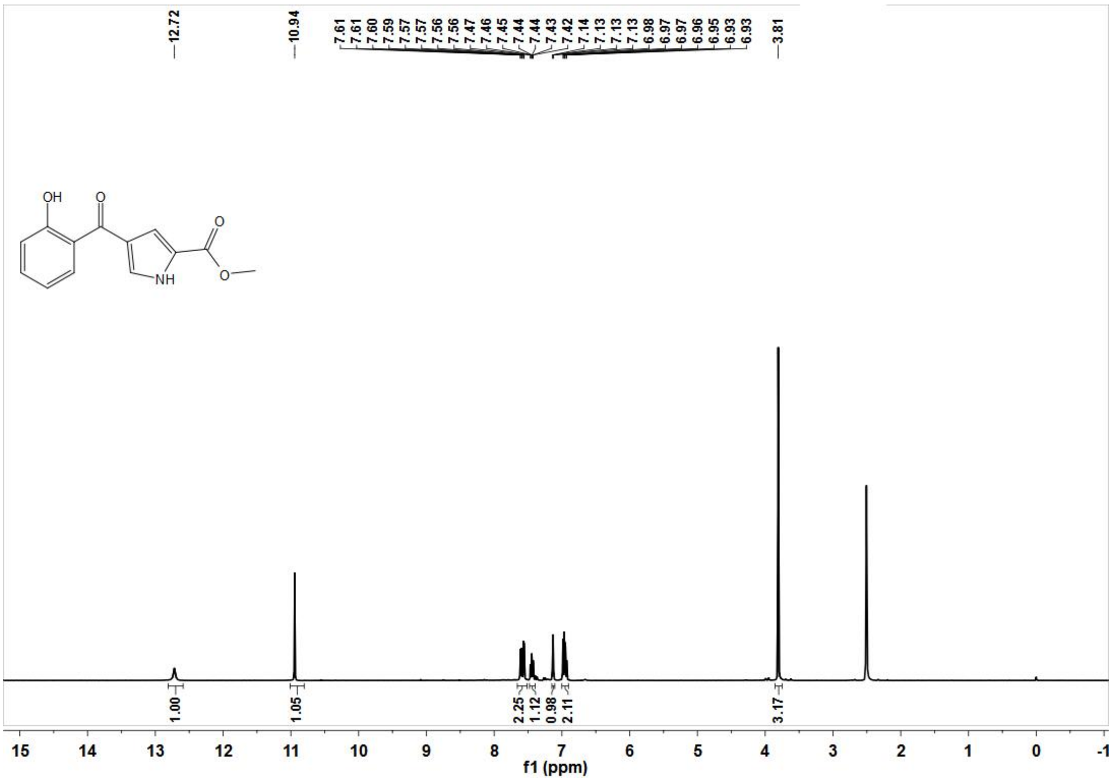

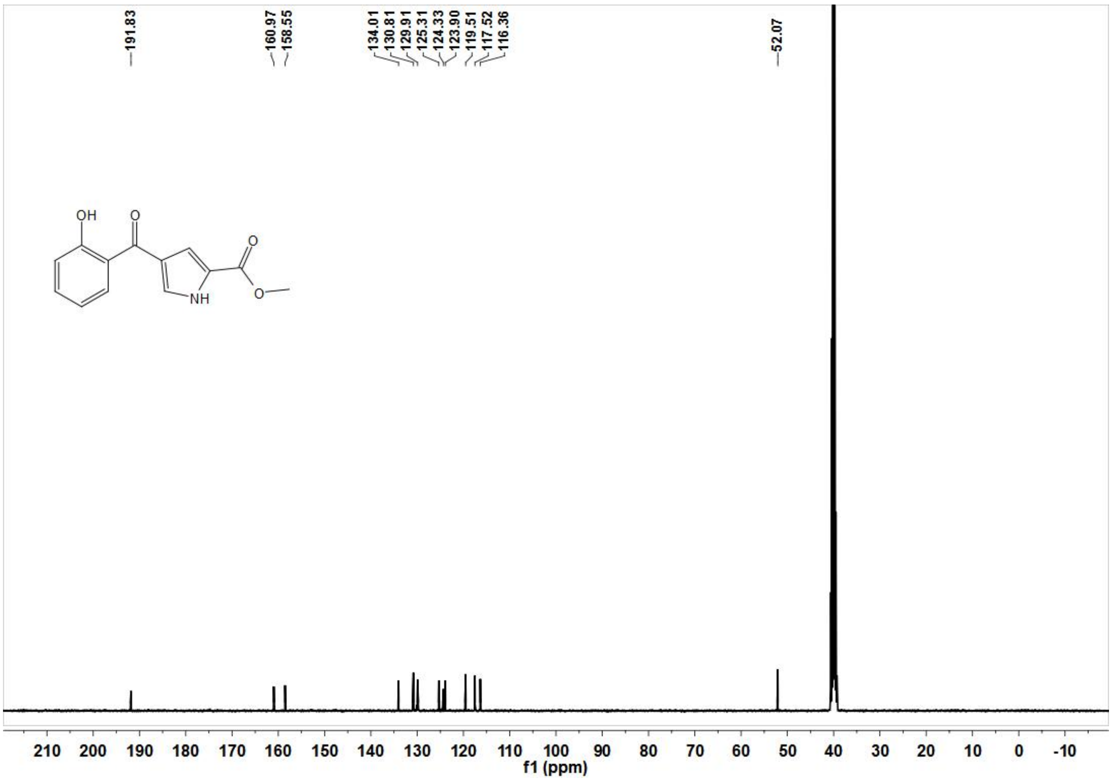

Figure S13. ^{1}H NMR and ^{13}C NMR spectra of **3m.**

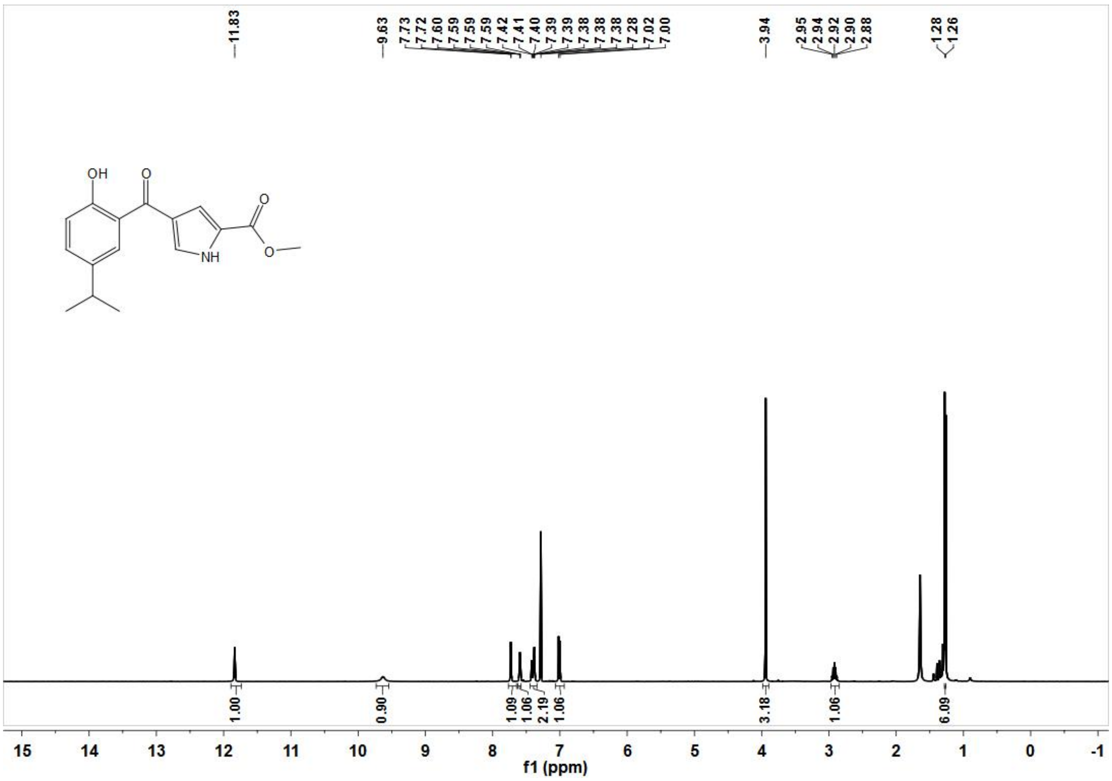

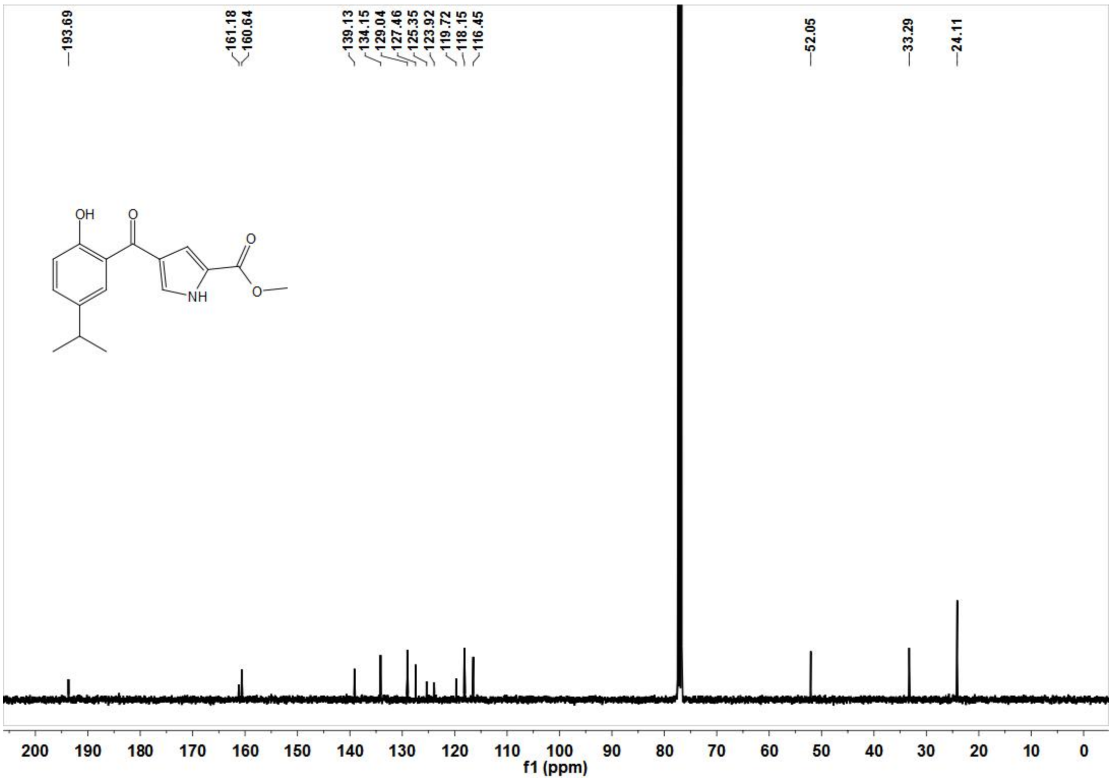

Figure S14. ^{1}H NMR, ^{13}C NMR and ^{19}F NMR spectra of **3n.**

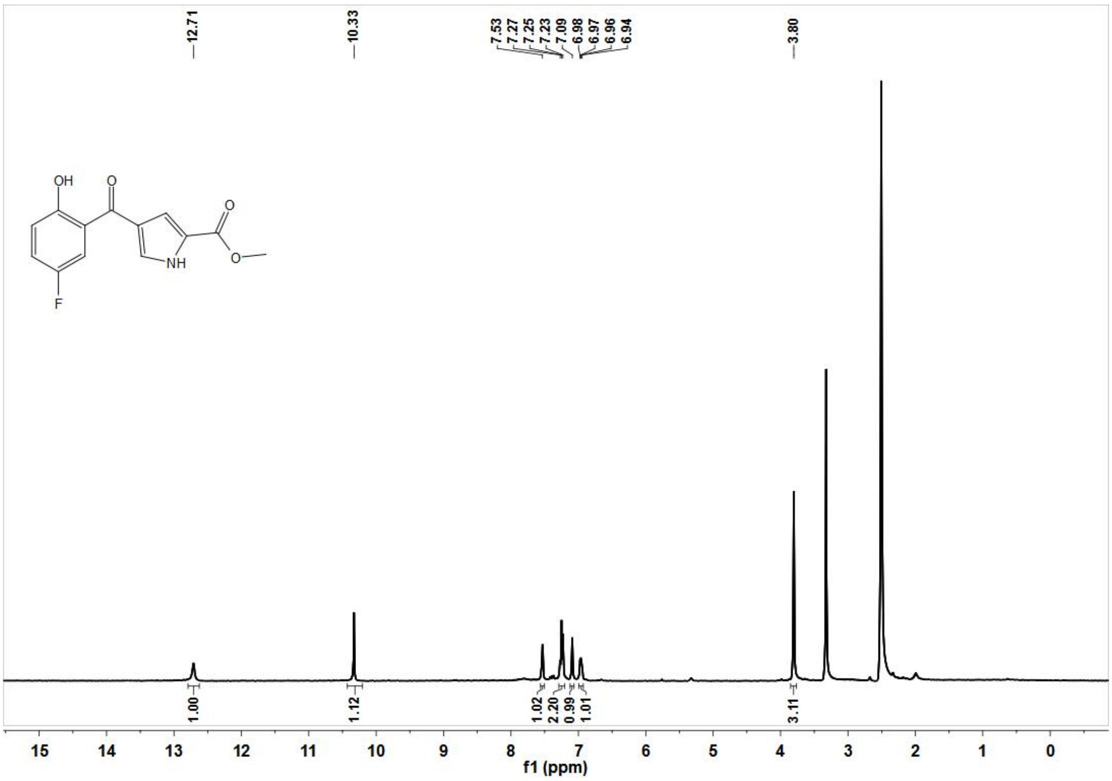

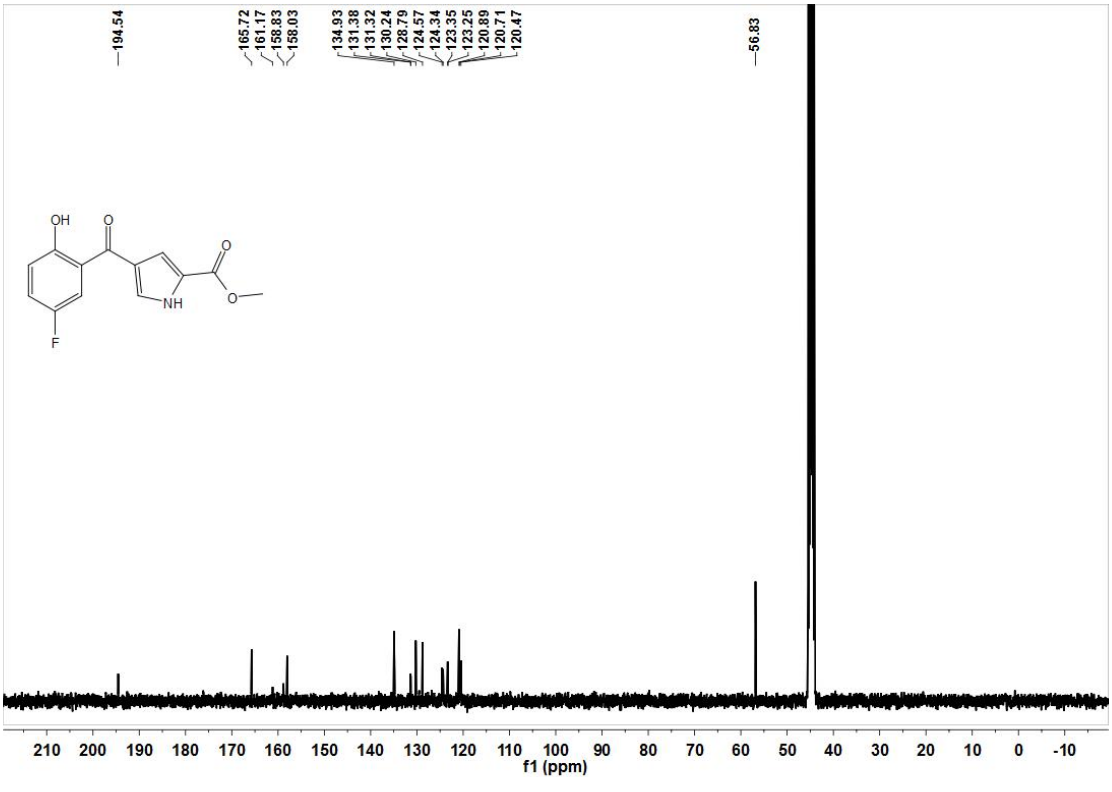

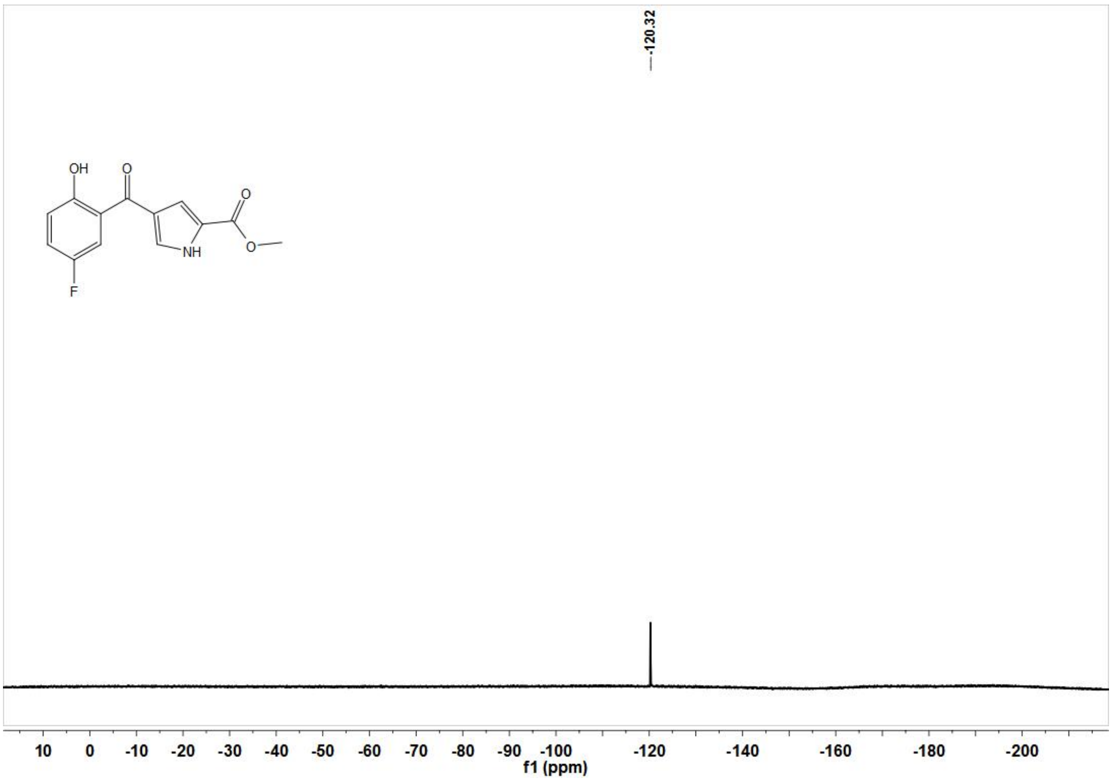
-120.32
OH
O
O
NH
O
F
10
0
-10
-20
-30
-40
-50
-60
-70
-80
-90
-100
-120
-140
-160
-180
-200
f1 (ppm)

Figure S15. ^{1}H NMR and ^{13}C NMR spectra of **3o.**

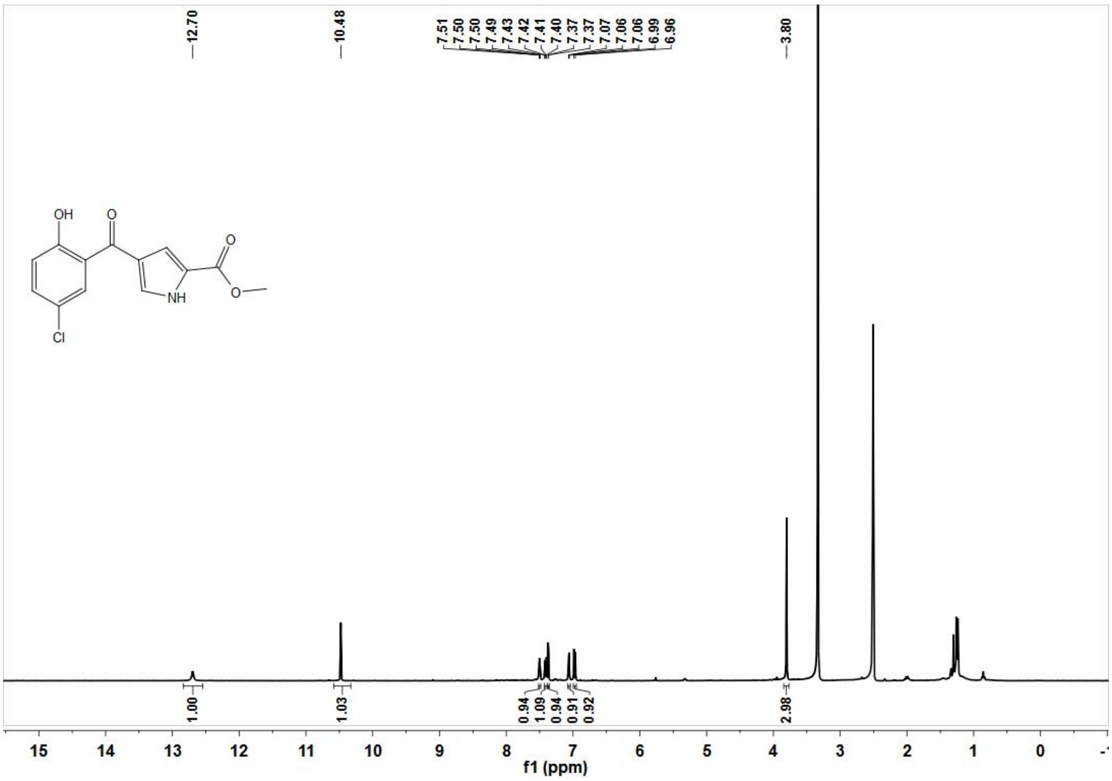

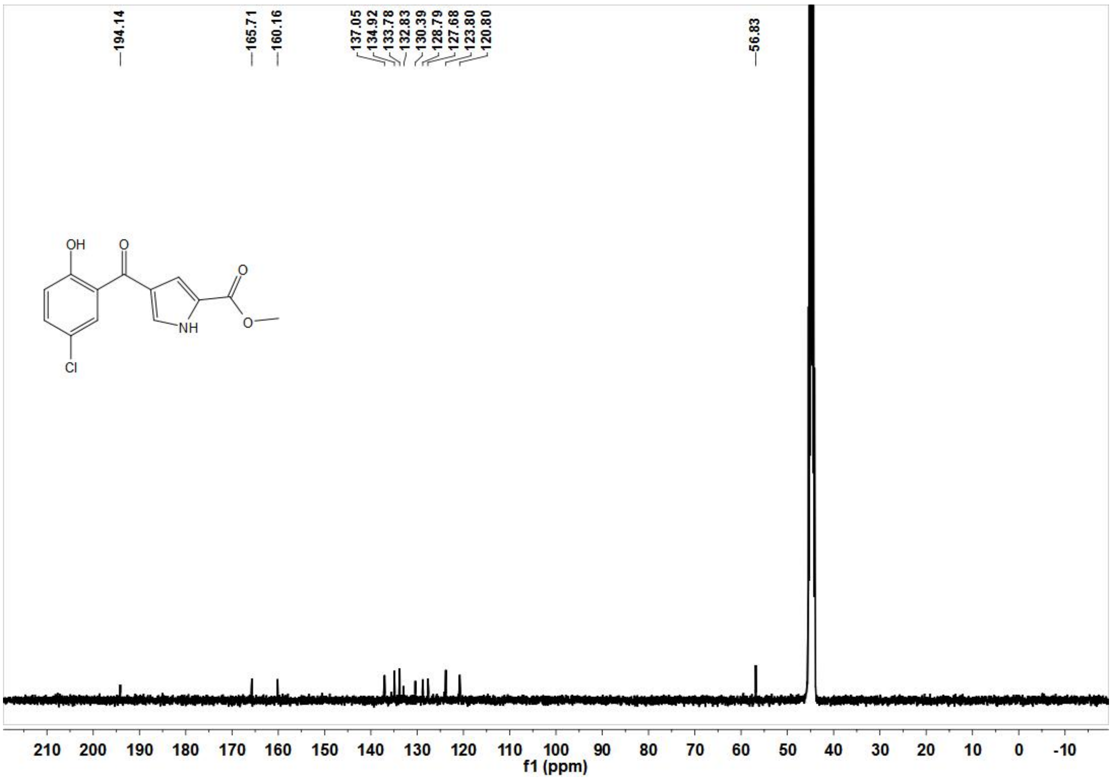

Figure S16. ^{1}H NMR and ^{13}C NMR spectra of **3p.**

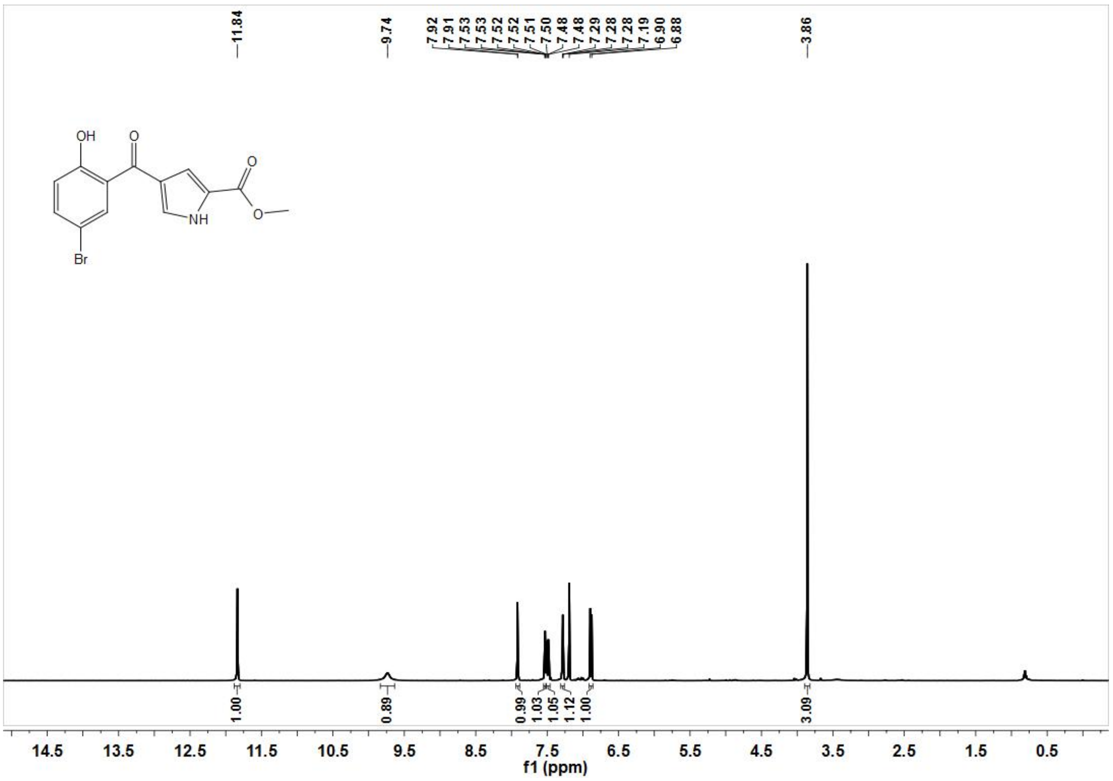

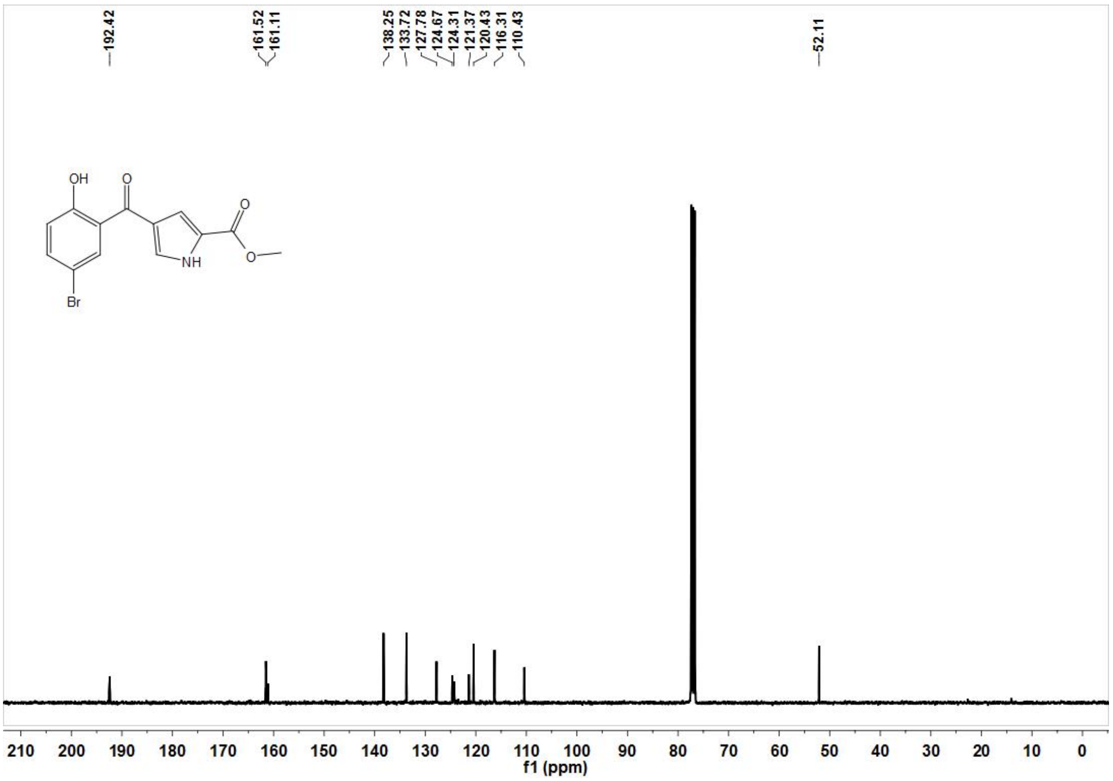

Supplement: Supplementary file 1 [file molecules-28-07602-s001.zip › molecules-2696677-supplementary-revised.pdf]
